# Clinical outcomes of advanced-stage glassy cell carcinoma of the uterine cervix: a need for reappraisal

**DOI:** 10.18632/oncotarget.12905

**Published:** 2016-10-25

**Authors:** Nara Yoon, Ji-Ye Kim, Hyun-Soo Kim

**Affiliations:** ^1^ Department of Pathology, The Catholic University of Korea Incheon St. Mary's Hospital, Incheon, Republic of Korea; ^2^ Department of Pathology, Severance Hospital, Yonsei University College of Medicine, Seoul, Republic of Korea

**Keywords:** cervix, glassy cell carcinoma, concurrent chemoradiation therapy, recurrence, survival

## Abstract

We performed a retrospective analysis of the clinical features and patient outcomes for advanced-stage glassy cell carcinoma of the uterine cervix. The study was restricted to cases in which the glassy cell features constituted at least 95% of the biopsied specimen. During the study period, 675 patients were diagnosed with primary cervical carcinoma. Five (0.7%) of the 675 patients had cervical glassy cell carcinoma; of these, three were premenopausal, and two were postmenopausal. Abnormal vaginal bleeding was the most frequent presenting symptom. Glassy cell carcinoma presented as a fungating, exophytic, or infiltrative mass. The greatest tumor dimension ranged from 3 to 9 cm. All patients had parametrial extension. Four patients had stage IIB tumors, and one had a stage IIIB tumor. All patients received concurrent chemoradiation therapy. The patient with a stage IIIB tumor died of hypovolemic shock caused by upper gastrointestinal bleeding during radiation therapy. Three patients with stage IIB tumors survived for more than 8 years without tumor recurrence or metastasis. One of these three patients died of pelvic recurrence 10 years after the initial diagnosis. Cervical glassy cell carcinoma has traditionally been considered an aggressive, highly malignant tumor with poor prognosis, but our data suggest that patient survival is not significantly decreased compared with other histological types of cervical carcinoma. It will be necessary to analyze patient outcomes using a larger number of cervical glassy cell carcinoma cases to confirm our findings.

## INTRODUCTION

Glassy cell carcinoma of the uterine cervix, accounting for approximately 1–2% of all cervical carcinomas, is a very rare form of cervical carcinoma [1–3]. According to the 2014 revised World Health Organization Classification of Tumours of Female Reproductive Organs, cervical glassy cell carcinoma is classified as a subtype of “other epithelial tumors” [4]. In 1956, Cherry and Glucksmann [5] first described cervical glassy cell carcinoma as a specific and distinctive entity of cervical carcinoma and classified this tumor as the most poorly differentiated adenosquamous carcinoma. They suggested sharp cytoplasmic margins; ground glass-appearing eosinophilic cytoplasm; and large, round to ovoid nuclei with prominent nucleoli as the morphological criteria for the diagnosis of glassy cell carcinoma. Two decades later, Littman et al. [2] described glassy cell carcinomas in detail, redefining and amplifying the morphological diagnostic criteria. According to two reports [2, 5], glassy cell features were defined based on the three main histopathological criteria: (1) cells with a moderate amount of ground-glass or finely granular cytoplasm that stains faintly blue with hematoxylin and eosin, (2) distinct cytoplasmic borders that stain with eosin and periodic acid-Schiff, and (3) large nuclei with conspicuous nucleoli. Since then, interest in glassy cell carcinoma has expanded, and many studies have been conducted [1–3, 6–21]; however, glassy cell carcinoma of the uterine cervix is still not clearly recognized. Moreover, due to its rarity, there have been no large cohort studies or clinical trials to clarify the outcomes of patients with this tumor or develop standard treatment strategies.

The clinical course of glassy cell carcinoma is contradictory to the data of prognosis and survival rates. Glassy cell carcinoma is refractory to conventional treatment modalities including radiotherapy and surgery [2]. Moreover, the reported clinical behavior of glassy cell carcinoma is aggressive, and this tumor is associated with a particularly poor prognosis due to rapid growth and frequent distant metastases [22–24]. However, some recent studies have suggested that glassy cell carcinoma might have a better prognosis than previously reported [1] and that patient survival is not significantly decreased compared with other histological types of cervical carcinoma [14].

We have treated several cases of advanced-stage cervical glassy cell carcinoma and recently reported their cytopathological characteristics [25]. The cytological specimens displayed amphophilic, granular tumor diathesis admixed with small clusters of tumor cells possessing large, round to oval nuclei and abundant, granular, ground-glass cytoplasm. The nuclei exhibited prominent eosinophilic nucleoli. In addition to these well-known morphological findings, we observed cytoplasmic molding and intercellular window formation between neighboring attached cells in liquid-based preparations. These characteristic cytomorphological features were confirmed on histopathological examination. During our previous study, we considered that it is necessary to further analyze the clinical features, treatment, and patient outcomes of this rare subtype of cervical carcinoma. The aim of this study was to describe the clinical characteristics and patient outcomes of cervical glassy cell carcinoma. Our results show that advanced-stage cervical glassy cell carcinoma responds well to concurrent chemoradiation therapy and that the overall survival rate of the patients is different from that reported previously, supporting the notion that glassy cell carcinoma patient survival is not significantly decreased compared with other histological types of cervical carcinoma.

## RESULTS

Brief case reports with detailed clinical information are provided below.

Case 1: A 38-year-old woman visited a local clinic with a complaint of irregular vaginal bleeding lasting for 1 month. Colposcopy revealed a fungating mass in the cervix, and she was referred to our institution. Magnetic resonance imaging (MRI) revealed a 3 × 2.5-cm fungating mass in the cervix, which extended to the right parametrium. The cytological diagnosis was carcinoma (type undetermined). A histopathological examination of a punch biopsy established a final diagnosis of glassy cell carcinoma. The patient remains alive without recurrence or metastasis 9 years after concurrent chemoradiation therapy.

Case 2: A 63-year-old female presented with heavy vaginal bleeding lasting for 1 hour. Colposcopy revealed a cervical mass, and she was referred to our institution. A pelvic examination revealed a 3-cm palpable cervical mass that extended to the right parametrium. MRI revealed an infiltrative mass located on the right side of the uterine cervix that had high signal intensity on a T2-weighted image. There was no evidence of enlarged lymph nodes or hydronephrosis. The cytological diagnosis was adenocarcinoma, and a histopathological diagnosis of glassy cell carcinoma was made after a punch biopsy. The patient remains alive without recurrence or metastasis 8 years after concurrent chemoradiation therapy.

Case 3: A 36-year-old female presented with postcoital vaginal bleeding. She had visited a local clinic because of vaginal bleeding that had first occurred 4 months previously. She had a prior history of pelvic inflammatory disease. She was prescribed antibiotics for suspected recurrent pelvic inflammatory disease, but the vaginal bleeding did not improve. She visited our institution because of repeated episodes of postcoital vaginal bleeding and left lower quadrant pain. Computed tomography (CT) revealed a 4-cm exophytic cervical mass extending to the bilateral parametrium. The cytological specimen was not suitable for evaluation due to the presence of excessive blood and inflammatory cells. A histopathological examination of a punch biopsy established a final diagnosis of glassy cell carcinoma. A sigmoid colostomy was performed because of a rectovaginal fistula that developed after initiation of concurrent chemoradiation therapy. The patient was under observation without any evidence of recurrence or metastasis, but MRI performed 9 years after diagnosis showed a large pelvic mass. The mass was located in the rectovaginal pouch and extended to the uterine cervix, perirectal soft tissue, rectum, vagina, parametrium, and urinary bladder. Multiple enlarged lymph nodes were also found in the bilateral inguinal areas and iliac chains. Liquid-based cytology of the recurred mass gave a diagnosis of glassy cell carcinoma. The patient died 1 year after tumor recurrence.

Case 4: A 67-year-old female visited our institution with edema and flank pain in the bilateral lower extremities. She had a history of heart failure. CT revealed bilateral hydronephrosis and extrinsic compression of the urinary bladder. Contrast-enhanced CT showed a 9 × 8-cm infiltrative mass involving the uterine cervix and corpus. This mass extended to the bilateral adnexae, parametrium, posterior bladder wall, and mesorectum. Multiple enlarged pelvic and para-aortic lymph nodes were present in the bilateral iliac chain and retroperitoneum, respectively. The imaging findings indicated that bilateral hydronephrosis had developed due to tumor-induced bilateral ureteral obstruction. Liquid-based cytology established a diagnosis of glassy cell carcinoma, which was confirmed histopathologically on a punch biopsy. The patient developed hematochezia due to upper gastrointestinal bleeding during concurrent chemoradiation therapy and was treated with conservative management but died of hypovolemic shock.

Case 5: A 37-year-old female presented with persistent vaginal bleeding for 2 months. Colposcopy revealed a 6 × 4-cm cervical polyp. Erosion and bleeding were found on the polyp surface. CT revealed an exophytic mass originating in the left cervical wall. The mass showed extensive hemorrhaging and necrosis with left parametrial extension. There was no evidence of enlarged lymph nodes or hydronephrosis. Two years after concurrent chemoradiation therapy, the patient remains alive without recurrence or metastasis.

Nine (1.3%) of the 675 cervical carcinoma patients in our study were diagnosed with glassy cell carcinoma or poorly differentiated adenosquamous carcinoma. Among the nine cases, we excluded one that showed areas of squamous differentiation (keratinizing squamous cell carcinoma), one that had areas of squamous and glandular differentiation (well-to-moderately differentiated adenosquamous carcinoma), and two that showed diffuse, strong p40 (squamous epithelial marker) expression. Therefore, the remaining five (0.7%) patients with cervical pure glassy cell carcinoma were included in a retrospective analysis, and their clinical features are summarized in Table 1. The median and mean patient ages at initial diagnosis were 38 and 48.2 years, respectively (range, 36-67 years). Three (60.0%) patients were premenopausal and younger than 40 years old; the remaining two (40.0%) were postmenopausal and older than 65. Two (40.0%) patients had significant previous medical histories. One (20.0%) patient had recurrent pelvic inflammatory disease, and another (20.0%) patient had hypertension and chronic heart failure. Abnormal vaginal bleeding was the most frequent presenting symptom noted in four (80.0%) patients. On imaging studies, glassy cell carcinomas presented as fungating, exophytic, or infiltrative tumor masses. The greatest tumor dimensions ranged from 3 to 9 cm.

**Table 1:** Summary of clinical features of cervical glassy cell carcinoma

Case	Age	PMHx	Symptom	Imaging finding	Greatest dimension	Location	PME	AdE	PWE	LNM	DM	HN	Stage
1	38	Absent	Irregular vaginal bleeding	Fungating mass	3 cm	Cervix	Present (right)	Absent	Absent	Absent	Absent	Absent	IIB
2	63	Absent	Heavy vaginal bleeding	Infiltrative mass	3 cm	Cervix	Present (right)	Absent	Absent	Absent	Absent	Absent	IIB
3	36	PID	Postcoital bleeding	Exophytic mass	4 cm	Cervix/Rectovaginal pouch*	Present (bilateral)	Absent	Absent	Absent/Present*	Absent	Absent	IIB
4	67	HTN, CHF	Leg edema, flank pain	Infiltrative mass	9 cm	Cervix	Present (bilateral)	Present (bilateral)	Absent	Present	Absent	Present (bilateral)	IIIB
5	37	Absent	Vaginal bleeding	Exophytic mass	6 cm	Cervix	Present (left)	Absent	Absent	Absent	Absent	Absent	IIB

Abbreviations: AdE: adnexal extension, CHF: congestive heart failure, DM: distant metastasis, HN: hydronephrosis, HTN: hypertension, LNM: lymph node metastasis, PMHx: previous medical history, PID: pelvic inflammatory disease, PME: parametrial extension, PWE: pelvic sidewall extension;

* At the time of recurrence

Regarding tumor extent, parametrial extension was observed in all patients, and adnexal extension was observed in one (20.0%) patient. One (20.0%) patient had multiple, bilateral pelvic and para-aortic lymph node metastases. There was no metastasis to distant organs at the time of initial diagnosis. Bilateral hydronephrosis was observed in one (20.0%) patient. Four (80.0%) patients had International Federation of Gynecology and Obstetrics stage IIB tumors, and the remaining case (20.0%) was a stage IIIB tumor. None of the patients were pregnant or had a recent history of pregnancy.

Pathological features, treatment, and outcomes of the five glassy cell carcinoma patients are summarized in Table 2. Based on pretreatment cytology, only one (20.0%) of the five cases was correctly diagnosed as glassy cell carcinoma. Three (60.0%) cases were diagnosed as carcinoma (type undetermined), adenocarcinoma, and poorly differentiated carcinoma, respectively. The remaining one (20.0%) case was interpreted as unsatisfactory for evaluation. The final histopathological diagnosis of glassy cell carcinoma was established in all cases. Human papillomavirus (HPV) genotyping revealed that all patients were infected with high-risk HPV. All patients received concurrent chemoradiation therapy; none underwent surgery. The median follow-up period was 8 years (range, 0.25-10 years). One (20.0%; case 4) patient with a stage IIIB tumor, as well as chronic heart failure and hypertension, died of hypovolemic shock caused by upper gastrointestinal bleeding during radiation therapy. Three (60.0%; cases 1, 2, and 3) patients with stage IIB tumors survived for more than 8 years without tumor recurrence or metastasis. One (case 3) of the three patients died of pelvic recurrence 10 years after initial diagnosis. Three (60.0%; cases 1, 2, and 5) of the five patients remain alive at the date of reporting.

**Table 2:** Summary of pathological features, treatment, and patient outcomes of cervical glassy cell carcinoma

Case	Cytology result	Biopsy result	HPV genotype	Treatment	Recurrence	RFS	OS	Current status
1	Carcinoma, type undetermined	Glassy cell carcinoma	High-risk (type 18)	CCRT	Absent	9 years	9 years	NED (currently alive)
2	Adenocarcinoma	Glassy cell carcinoma	High-risk (type 16)	CCRT	Absent	8 years	8 years	NED (currently alive)
3	Unsatisfactory/Glassy cell carcinoma*	Glassy cell carcinoma	High-risk (type 31)	CCRT	Present	9 years	10 years	DOD
4	Glassy cell carcinoma	Glassy cell carcinoma	High-risk (type 18)	CCRT	Absent	3 months	3 months	DOO
5	Poorly differentiated carcinoma	Glassy cell carcinoma	High-risk (type 18)	CCRT	Absent	2 years	2 years	NED (currently alive)

Abbreviations: CCRT: concurrent chemoradiation therapy; DOD: dead of tumor-related disease; DOO: dead of other causes; HPV: human papillomavirus; OS: overall survival; RFS: recurrence-free survival;

* At the time of recurrence

**Table 3:** Histopathological diagnosis in 675 patients with primary cervical carcinoma

Histopathological diagnosis	Number of cases (%)
Squamous cell carcinoma	407 (60.3%)
Endocervical adenocarcinoma, usual type	104 (15.4%)
Endocervical adenocarcinoma *in situ*	47 (7.0%)
Squamous cell carcinoma *in situ*	46 (6.8%)
Poorly differentiated carcinoma	28 (4.1%)
Mucinous carcinoma	24 (3.6%)
High-grade neuroendocrine carcinoma	8 (1.2%)
Glassy cell carcinoma	5 (0.7%)
Serous carcinoma	4 (0.6%)
Clear cell carcinoma	2 (0.3%)
Total	675 (100.0%)

## DISCUSSION

Due to the rarity of cervical glassy cell carcinoma, it is debatable whether it is a distinct clinicopathological entity and separate histological type. Although the World Health Organization Classification defines glassy cell carcinoma as a poorly differentiated variant of adenosquamous carcinoma, several reports have stated that glassy cell carcinoma should be considered the most poorly differentiated variant of adenosquamous carcinoma without obvious differentiation toward any specific lineages [1, 11, 14, 23]. The histopathological features of glassy cell carcinoma are similar to those of poorly differentiated squamous cell carcinoma, poorly differentiated adenocarcinoma, and adenosquamous carcinoma. When glassy cell carcinoma is suspected, it is necessary to determine whether there are areas of differentiation into a certain lineage. If there is a tendency toward squamous differentiation (keratin pearls, individual keratinization, intercellular bridges, or strong p40 immunoreactivity) and/or glandular differentiation (acinar architecture, nuclear polarization, rudimentary glands with mucin production, or strong immunoreactivity for carcinoembryonic antigen or mucin), a diagnosis should be made according to the corresponding histological type (squamous cell carcinoma, adenocarcinoma, or adenosquamous carcinoma). Unfortunately, there have been no established quantitative criteria for glassy cell carcinoma, and it remains unclear what percentage of area showing glassy cell features is required for the diagnosis. This study was restricted to cases in which glassy cell features (sharp cytoplasmic margins; ground glass-appearing eosinophilic cytoplasm; and large, round to ovoid nuclei with prominent nucleoli) constituted at least 95% of the specimen.

Glassy cell carcinoma of the cervix has been associated with a poorer prognosis than that of other histological subtypes of cervical carcinoma [4]. In 1976, Littman et al. [2] published the first series of 13 patients with glassy cell carcinoma, reporting an overall survival rate of 31%. Subsequent series also noted similar poor outcomes. Prior to 2000 [2, 16, 23, 26–28], the 5-year overall survival rate was approximately 50% [1]. Moreover, the 5-year overall survival rate of stage I glassy cell carcinoma patients was approximately 64% [1], which is much lower than that for all stage I cervical carcinomas (80-93%). However, recent studies have not confirmed the previous reports [1, 14, 29]. In 2002, Gray et al. [1] investigated outcomes of 22 glassy cell carcinoma patients, and the overall survival rate for all stages was 73%, which was comparable with the 75% survival rate of all cervical carcinomas and higher than the previously described survival rate [29]. Similarly, the 5-year overall survival rates for stage I glassy cell carcinoma reported by Gray et al. [1] and Guitarte et al. [29] were 86% and 73.5%, respectively, both of which were comparable with the overall survival for all stage I cervical carcinomas [30]. Earlier studies also found that the majority of glassy cell carcinomas are diagnosed at stage I, and this stage distribution is similar to that of squamous cell carcinoma [29]. It is inferred that the improvement in overall survival might be due to advances in imaging and surgical techniques leading to earlier glassy cell carcinoma detection. In this study, the 5-year overall survival rate for the three patients with stage IIB glassy cell carcinoma was 100%. Although it is difficult to determine the reasons for such excellent survival rates because of the small number of cases, concurrent chemoradiation therapy appeared to improve patient outcome for stage II disease [29]. The previously reported recurrence rates of glassy cell carcinoma were 33%, 3%, and 0% for patients treated with surgery, radiotherapy, and chemoradiation therapy, respectively [29]. The clinical behavior of glassy cell carcinoma, including treatment response, is difficult to characterize given limited information on staging and treatment from previous studies, which had small case numbers and various management approaches. There is a crucial need for retrospective multicenter evaluations of glassy cell carcinoma treatments to expand knowledge about this rare entity.

In conclusion, we described the clinical characteristics and patient outcomes of cervical glassy cell carcinoma. Three patients with advanced disease who underwent concurrent chemoradiation therapy survived for more than 8 years without evidence of recurrence or metastasis, suggesting that survival following a diagnosis of advanced-stage cervical glassy cell carcinoma is not significantly decreased compared with that for other histological subtypes of cervical carcinoma.

## MATERIALS AND METHODS

During the study period from July 2007 to June 2016, a total of 675 patients were diagnosed with primary cervical carcinoma. Histopathological diagnoses of the cases we collected during the study period are summarized in Table 3. Of the 675 patients, 407 (60.3%) and 104 (15.4%) patients had invasive squamous cell carcinoma and endocervical adenocarcinoma (usual type), respectively. Endocervical adenocarcinoma *in situ* and squamous cell carcinoma *in situ* were detected in 47 (7.0%) and 46 (6.8%) patients, respectively. Thirty-three (4.9%) patients were diagnosed with poorly differentiated carcinoma of the uterine cervix. The others were diagnosed with mucinous carcinoma (24, 3.6%), high-grade neuroendocrine carcinoma (8, 1.2%), serous carcinoma (4, 0.6%), or clear cell carcinoma (2, 0.3%). Of the 33 cervical poorly differentiated carcinomas, 9 cases were diagnosed as glassy cell carcinoma (3, 0.4%) or poorly differentiated adenosquamous carcinoma (6, 0.9%). We reviewed all available hematoxylin and eosin-stained slides obtained from the nine cases. Five cases of cervical glassy cell carcinoma were finally selected.

Pretreatment cytology and punch biopsy specimens were available for all cases. Primary cervical carcinoma was histopathologically classified according to the 2014 revised World Health Organization of Tumours of Female Reproductive Organs [4]. The International Federation of Gynecology and Obstetrics staging system was applied to determine tumor stage [21, 30]. Clinical and pathological information were obtained from the electrical medical information systems and pathology reports. The clinical details that were reviewed included patient age; previous medical history; presenting symptom; greatest tumor dimension; presence of parametrial, adnexal, and/or pelvic sidewall extension; presence of lymph node metastasis; presence of distant metastasis; presence of hydronephrosis; stage; treatment; recurrence-free and overall survival times; and current status. This study was reviewed and approved by the Institutional Review Board of Severance Hospital, Yonsei University Health System, Seoul, Republic of Korea (2016-1010-001).

For liquid-based cytological smears, samples were taken from the fornix, portio, and endocervix. The ThinPrep test (Hologic Inc., Marlborough, MA, USA) was performed using an automated liquid-based monolayer cell preparation system (ThinPrep 2000 system; Hologic Inc.) as previously described [25]. The biopsied specimens were fixed in 10% neutral-buffered formalin and embedded in paraffin blocks. From each formalin-fixed, paraffin-embedded block, 4-μm sections were cut and stained with hematoxylin and eosin. A variable number of hematoxylin and eosin-stained slides from each case were available for review. We chose the most representative slide containing an appropriate volume of tumor and possibly normal cervical tissue for immunohistochemical staining and HPV genotyping. Immunohistochemical staining was performed using the Ventana Benchmark XT automated staining system (Ventana Medical Systems, Tucson, AZ, USA) or the Dako Omnis (Dako, Agilent Technologies, Carpinteria, CA, USA), as previously described [25, 31, 32]. We also performed polymerase chain reaction-based microarray for HPV genotyping using a commercially available HPV 9G DNA chip (BMT HPV 9G DNA Chip; Biometrix Technology, Chuncheon, Republic of Korea), as previously described [25, 31].
